# Frequency Dependent Topological Patterns of Resting-State Brain Networks

**DOI:** 10.1371/journal.pone.0124681

**Published:** 2015-04-30

**Authors:** Long Qian, Yi Zhang, Li Zheng, Yuqing Shang, Jia-Hong Gao, Yijun Liu

**Affiliations:** 1 Department of Biomedical Engineering, Peking University, Beijing, China; 2 School of Life Science and Technology, Xidian University, Xi’an, Shaanxi, China; 3 Department of Biological Sciences, National University of Singapore, Singapore, Singapore; 4 Center for MRI Research, Peking University, Beijing, China; Max Planck Institute for Human Cognitive and Brain Sciences, GERMANY

## Abstract

The topological organization underlying brain networks has been extensively investigated using resting-state fMRI, focusing on the low frequency band from 0.01 to 0.1 Hz. However, the frequency specificities regarding the corresponding brain networks remain largely unclear. In the current study, a data-driven method named complementary ensemble empirical mode decomposition (CEEMD) was introduced to separate the time series of each voxel into several intrinsic oscillation rhythms with distinct frequency bands. Our data indicated that the whole brain BOLD signals could be automatically divided into five specific frequency bands. After applying the CEEMD method, the topological patterns of these five temporally correlated networks were analyzed. The results showed that global topological properties, including the network weighted degree, network efficiency, mean characteristic path length and clustering coefficient, were observed to be most prominent in the ultra-low frequency bands from 0 to 0.015 Hz. Moreover, the saliency of small-world architecture demonstrated frequency-density dependency. Compared to the empirical mode decomposition method (EMD), CEEMD could effectively eliminate the mode-mixing effects. Additionally, the robustness of CEEMD was validated by the similar results derived from a split-half analysis and a conventional frequency division method using the rectangular window band-pass filter. Our findings suggest that CEEMD is a more effective method for extracting the intrinsic oscillation rhythms embedded in the BOLD signals than EMD. The application of CEEMD in fMRI data analysis will provide in-depth insight in investigations of frequency specific topological patterns of the dynamic brain networks.

## Introduction

Human brain is considered as a large-scale complex network endowed with a small-world architecture which is characterized by a high-level local connectedness and an exceedingly short path length linking individual network nodes [1]. A quantitative analysis of the complex brain networks, largely based on graph theory analysis, is typically achieved through all major magnetic resonance imaging (MRI) modalities and neurophysiological data from both functional and structural perspectives [2]. Under this framework, resting-state functional MRI (fMRI), a non-invasive way of measuring the spontaneous neural activities in the human brain, has been widely applied to investigate the fundamental topological organization of brain networks [3]. In addition, recent studies based on resting-state fMRI [4–8] have revealed the associations between the topological organization of brain networks and cognitive performance or psychiatric brain disorders respectively, which suggest that resting-state fMRI may provide new approaches to assess brain network properties in both healthy and diseased brains.

Notably, most previous studies related to brain networks [1, 3] have concentrated on the conventional frequency band (0.01–0.1 Hz) derived from resting-state fMRI. It is demonstrated that frequency specificities of functional connectivity (FC) [9, 10], regional homogeneity (ReHo) [11], amplitude of low frequency fluctuations (ALFF) [12] or energy [13, 14] existed extensively within the fluctuations of blood oxygenation level-dependent (BOLD) signals. Lacking the physiological explanation and definition of various frequency bands in BOLD signals, however, the distinct spatial distribution of fluctuations in various frequency ranges still indicate the non-trivial potential application of mapping the human brain from the perspective of frequency based on resting-state fMRI.

Accompanied by increasing attention of frequency specificities in BOLD signals, partial results in previous studies [15–18] have suggested that the distinct or invariant topological patterns are distributed in various frequency intervals. The frequency specificities in small world networks have been delineated in the previous studies [15–18], however, the detailed frequency dependent topological patterns remain concealed. Particularly, the global but not regional topological patterns are investigated across different frequency bands (Salvador *et al*. [18], Achard *et al*.[15] and Supekar *et al*.[17]); and the maximal frequency band applied is no more than 0.073 Hz (Liang *et al*.[16]).

In the past decade, a number of studies have attempted to delineate the frequency specificities of BOLD signals using Wavelet or Fourier transformation or simple ordinary band-pass filter [10, 13, 15]. However, the inherent assumptions of linearity in a Wavelet analysis or linearity and stationarity in a Fourier analysis may impose limitations on the findings, since the assumptions have not been verified in the BOLD time series [19]. Moreover, the full band width has been decomposed into small bands arbitrarily and without rigorous justifications [11]. On the other hand, we have previously [11] introduced a data driven method named empirical mode decomposition (EMD) (Huang *et al*. [20]) to adaptively decompose the whole brain BOLD time series into several intrinsic mode functions (IMFs). A distinctive frequency range is occupied by each IMF component: the highest frequency range is occupied by the first IMF, and the lowest frequency interval by the last IMF, with the remaining ones in between respectively. EMD can overcome the limitations of the Wavelet or Fourier transformation, since it implies no assumption of linearity, stationarity, or recourse to any prior rigid chosen band-pass filter [20]. However, the phenomenon of mode-mixing induced by intermittence signals is a troublesome issue in EMD [21]. To overcome such deficit, the improved data driven method named complementary ensemble empirical mode decomposition (CEEMD) was proposed in the current study [22]. CEEMD is modified from a noise-assisted ensemble empirical mode decomposition (EEMD) [21], which can resolve the mode-mixing problem and effectively eliminate the residue noise in each IMF [21, 22].

To investigate the frequency characteristics of resting state brain networks, CEEMD was applied in a voxel-wise fashion to adaptively decompose the time series of each voxel into several IMFs. It is hypothesized that CEEMD may perform better in the elimination of the mode-mixing effect emerged in EMD algorithm. Thereafter, both the global and nodal topological connectivity properties were investigated to reveal the organization of frequency specific brain networks. Furthermore, the reliability of CEEMD was verified using a split-half analysis to test the reproducibility of the results; and a conventional ideal rectangular window band-pass filter was applied to divide the BOLD oscillations into the same frequency bins defined by CEEMD. Comparison was performed between the two methods.

## Materials and Methods

### MRI Data Acquisition

The current research was approved by the Institutional Review Board of Peking University. MRI data was obtained from an open source website (http://fcon_1000.projects.nitrc.org/fcpClassic/FcpTable.html) provided by the ‘1000 Functional Connectomes’ Project. Data (122 females, 76 males, age range: 18–26 years old) was collected by Beijing Normal University and analyzed in the current study. All of the subjects had no history of psychiatric disorders, or any neurological illness. Informed written consent forms were obtained from each participant prior to scanning in accordance with Institutional Review Board guidelines of Beijing Normal University and in compliance with the Declaration of Helsinki.

All data were acquired on a 3.0Tesla MR system (Siemens 3T Trio). All subjects were required to rest with their eyes closed, with no particular thoughts during the scan and they were asked not to fall asleep. A gradient echo T2*-weighted EPI sequence was applied to acquire resting state functional images with the following parameters: TR = 2000 ms, TE = 30 ms, flip angle = 80°; matrix size = 64 × 64, FOV = 240 × 240 mm^2^, which gave an in-plane resolution of 3.75 mm × 3.75 mm, 51 axial slices (3.5 mm thickness with a gap of 1.2 mm). The scan of resting state fMRI lasted 450 seconds, covering 225 brain volumes. In addition, a T1-weighted three-dimensional magnetization prepared rapid gradient echo (MPRAGE) sequence was acquired that covered the entire brain, with the following parameters: 128 slices, TR = 2530 ms, TE = 3.39 ms, flip angle = 7°, inversion time = 1100 ms, FOV = 256 × 256 mm and in-plane resolution = 256 × 256.

### Image Preprocessing

Images were analyzed by using both the FMRIB Software Library (FSL: http://www.fmrib.ox.ac.uk/fsl, version 5.0) and Analysis of Functional NeuroImaging (AFNI: http://afni.nimh.nih.gov/afni, version 2011_12_21_1014). The main preprocessing included the following steps: (1) the removal of the first 10 time points for the signal steady state and for the adaptation of the participants to the environment (AFNI: 3dcalc); (2) the correction for the difference in image acquisition time among the slices (AFNI: 3dTshift) and head motion during data acquisition (AFNI: 3dvolreg) on the remaining 215 volumes of the functional BOLD images. Hence, the mean image was acquired by averaging the volumes of each subject (AFNI: 3dTstat). Subjects with translational or rotational parameters exceeding ± 1 mm or ± 1° in their data were excluded, therefore, 161 subjects were eventually included in the analysis; (3) the co-registration of the individual structural image to the mean functional image by using a linear transformation (FSL: flirt) and the estimation of a nonlinear transformation from individual space of the co-registered structural image into MNI152 space (FSL: flirt and fnirt); (4) spatial normalization of the functional image to a standard template (Montreal Neurological Institute) by using the normalization parameters estimated in the last procedure (FSL: applywarp), resulting in a functional image series of 61×73×61 voxels (3-mm isotropic voxels). (5) the performance of a regression of nuisance variables (including white matter, ventricular signals, global signals and the six motion parameters determined in the realignment procedure) from the data to reduce the influence of motion and unspecific physiological effects (AFNI: 3dDeconvolve), but not spatially smoothed as previously suggested [11]; and (6) the regression of the linear trend from the time course of each voxel to remove signal drifts caused by scanner instability or other sources (AFNI: 3dTcat).

### Complementary ensemble empirical mode decomposition

CEEMD originates from EMD invented by Huang [20] and extended from ensemble empirical mode decomposition (EEMD) by Wu [21]. The detailed description of EMD was presented in the supplementary materials. The EEMD generates an ensemble of data sets by adding different realizations of a white noise with finite amplitude *ε*0 to the original data. EMD analysis is then applied to each data series of the ensemble; ultimately, the IMFs are achieved by averaging the respective components in each realization over the ensemble [21]. The averaging effect of the assisted white noise *εf* decreases as:

$$\varepsilon f=\varepsilon /\sqrt{NE}$$ (1)

In Eq 1, *ε* = *ε*0*std*(y0) and *ε*0 is the input noise level, where *y*0 represents the input signal and NE is the ensemble number. Theoretically, NE approaches infinity in order to smooth out the assisted white noise. In practice, *ε*0 is selected in the interval of 0.1–0.4; NE of the order of 100 will generally produce satisfactory results and render the residual noise less than a fraction of 1% of the error [21].

To further reduce the white noise residue in each IMF component and time consumption, CEEMD was applied here, where white noise was particularly included in pairs to the original data (i.e. one positive and one negative) to generate two sets of ensemble IMFs [22]. Additionally, to visualize the frequency distribution of each IMF component, Hilbert weighted frequency (HWF) of each IMF [23] was applied to reflect the mean oscillation frequency of the IMF [11].

### Graph Analysis

#### Network Construction

In the current study, the automated anatomical labeling (AAL) template image was applied for regional parcellation approach as previously validated by Tzourio-Mazoyer [24]. Thus, each hemisphere was divided into 45 anatomical regions of interest (ROIs), which are listed in Table 1 together with the abbreviated regional labels. Regional mean time series were estimated for each subject across five IMFs by averaging the fMRI time series over all voxels in each of the 90 regions. Pearson correlation coefficient was performed to estimate the IMF dependent correlations between each of the 4005 possible pairs of the 90 cortical and subcortical (90 ROI from the common AAL atlas) BOLD signals derived from each individual set. A set of five (90×90) inter-regional Pearson correlation matrices were then obtained for each subject. False discovery rate (FDR) correction was applied to regulate the expected FDR at the statistical significance threshold as 0.05 in individual level. Thus, five frequency dependent population-based functional connectivity networks were constructed by capturing the underlying common connectivity pattern of the brain.

**Table 1:** Cortical and subcortical regions of interest defined in study.

Index	Region	Abbr.	Index	Region	Abbr.
(1,2)	Precental gyrus	PreCG	(47,48)	Lingual gyrus	LING
(3,4)	Superior frontal gyrus, dorsolateral	SFGdor	(49,50)	Superior occipital gyrus	SOG
(5,6)	Superior frontal gyrus, orbital part	ORBsup	(51,52)	Middle occipital gyrus	MOG
(7,8)	Middle frontal gyrus	MFG	(53,54)	Inferior occipital gyrus	IOG
(9,10)	Middle frontal gyrus, orbital part	ORBmid	(55,56)	Fusiform gyrus	FFG
(11,12)	Inferior frontal gyrus, opercular part	IFGoperc	(57,58)	Postcentral gyrus	PoCG
(13,14)	Inferior frontal gyrus, triangular part	IFGtriang	(59,60)	Superior parietal gyrus	SPG
(15,16)	Inferior frontal gyrus, orbital part	ORBinf	(61,62)	Inferior parietal, but supramarginal and angular gyri	IPL
(17,18)	Rolandic operculum	ROL	(63,64)	Supramarginal gyrus	SMG
(19,20)	Supplementary motor area	SMA	(65,66)	Angular gyrus	ANG
(21,22)	Olfactory cortex	OLF	(67,68)	Precuneus	PCUN
(23,24)	Superior frontal gyrus, medial	SFGmed	(69,70)	Paracentral lobule	PCL
(25,26)	Superior frontal gyrus, medial orbital	ORBsupmed	(71,72)	Caudate nucleus	CAU
(27,28)	Gyrus rectus	REC	(73,74)	Lenticular nucleus, putamen	PUT
(29,30)	Insula	INS	(75,76)	Lenticular nucleus, pallidum	PAL
(31,32)	Anterior cingulate and paracingulate gyri	ACG	(77,78)	Thalamus	THA
(33,34)	Median cingulate and paracingulate gyri	DCG	(79,80)	Heschl gyrus	HES
(35,36)	Posterior cingulate gyrus	PCG	(81,82)	Superior temporal gyrus	STG
(37,38)	Hippocampus	HIP	(83,84)	Temporal pole: superior temporal gyrus	TPOsup
(39,40)	Parahippocampal gyrus	PHG	(85,86)	Middle temporal gyrus	MTG
(41,42)	Amygdala	AMYG	(87,88)	Temporal pole: middle temporal gyrus	TPOmid
(43,44)	Calcarine fissure and surrounding cortex	CAL	(89,90)	Inferior temporal gyrus	ITG
(45,46)	Cuneus	CUN			

The regions are listed according to a prior template obtained from an AAL atlas. Odd numbers represent the corresponding brain regions in the left hemisphere, and even numbers denote the specific brain regions in the right hemisphere.

#### Network Analysis

*Global topological parameters*: Previous studies [25, 26] have demonstrated two key metrics applied to describe the complex networks in human brain: clustering coefficient (Cp) and characteristic path length (Lp). In order to investigate the small-world properties, Cp and Lp should be compared to the corresponding random networks [27]. In general, a small-world network should have significantly higher clustering coefficient value than that of random network (γ = Cp_(real)_/Cp_(rand)_>1) while the characteristic path length being approximately equivalent compared with random networks (λ = Lp_(real)_/Lp_(rand)_∼1) [28]. These two measurements can be summarized into simple quantitative metric, small-worldness, *σ* = *γ*/*λ*, which is typically greater than 1 for small-world networks [25, 26]. In the current study, weighted degree, S^w^(G), global efficiency, E_glob_(G), and local efficiency, E_loc_(G), of a network G were investigated as well. The weighted degree of a network refers to the average of the edge weights linking to a specific node across all of the nodes [29]. The E_glob_(G) measures the global efficiency of the parallel information transfer in the network, while E_loc_(G) reveals the fault tolerance of the network. It indicates the efficiency of the communication between the first neighbors of i, if i is removed [26]. In order to observe these frequency specificities of small world networks, the graph characteristics were calculated at multi-sparsity (or density), which represented the fraction of present connections to all possible connections [30]. Notably, in the current study, the estimation of all the parameters were under consideration of weighted coefficient, which was consistent with the previous study [31]. The estimation of all parameters were calculated using the code provided by Brain Connectivity Toolbox (BCT) [32].

*Regional nodal characteristics*: Three nodal topological characteristics were applied, including nodal weighted degree ${(\text{S}}_{\text{i}}^{\text{w}})$, nodal global efficiency ${(\text{E}}_{\text{i},\text{g}\text{l}\text{o}\text{b}}^{\text{w}})$ and nodal betweenness ${(\text{B}}_{\text{i}}^{\text{w}})$. Nodal weighted degree is computed as the sum of the weight of all the connections of node i, which quantifies the extent to which a node is relevant to the graph [32]. The nodal efficiency of a given node ${\text{E}}_{\text{i},\text{g}\text{l}\text{o}\text{b}}^{\text{w}}$ is defined as the inverse of the mean harmonic shortest path length between the node of interest and all the remaining nodes in the network [33], which quantifies the importance of the nodes for the communication within the network [33]. The betweenness centrality of a node i considers the fraction of all shortest paths in the network that pass through the node [34], which captures the influence of a node over information flow between other nodes in the network. To investigate the frequency specificities of hub distribution, AUC of each nodal measure ${\text{(S}}_{\text{i}}^{\text{w}}{\text{, E}}_{\text{i,glob}}^{\text{w}}\text{,}{\text{ B}}_{\text{i}}^{\text{w}}\text{)}$ was selected across a range of interested densities in all IMF components as the estimation for each node, denoted as ${\text{S}}_{\text{i}}^{\text{(w,auc)}}{\text{, E}}_{\text{i,glob}}^{\text{(w,auc)}}{\text{, B}}_{\text{i}}^{\text{(w,auc)}}$ respectively. Due to the absence of a formal consensus regarding selection of thresholds, here we selected a range of sparsity threshold (S_min_(0.14)≤ sparsity≤0.40) for functional connectivity networks, where S_min_ represented a minimum network sparsity in which all nodes would become fully connected in the five IMF-dependent brain networks [30]. The nodes representing the high ${\text{S}}_{\text{i}}^{\text{(w,auc)}}{\text{, E}}_{\text{i,glob}}^{\text{(w,auc)}}{\text{, B}}_{\text{i}}^{\text{(w,auc)}}$ were considered as the hubs (no less than one standard deviation (SD) larger than the average nodal AUC values of the network) [30].

### Reproducibility Analysis

To investigate the reproducibility of the results, a split-half analysis was performed [31]. Two independent age- and gender-matched subgroups were created (80 and 81 participants). For each subgroup, the frequency specific brain networks were separately constructed and analyzed respectively using the same methods as the aforementioned whole-group analysis. The results of the two subgroups were compared to evaluate the reproducibility. In addition, to further verify the reliability of CEEMD and the accuracy of frequency intervals defined by CEEMD and HWF, the BOLD signals were filtered to the same five specific frequency bands, which were defined by CEEMD and HWF, by using filter functions provided in the REST toolbox [35]. For convenience, this comparison method was denoted as “REST”.

## Results

### Frequency distribution and IMF dependency of FC

**Fig 1:**
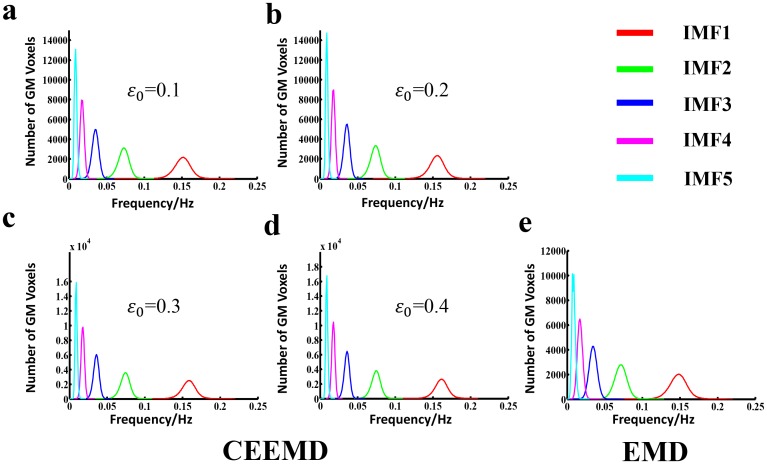
Histogram of frequency distribution using CEEMD and EMD, respectively. From Fig 1a to Fig 1e, each figure represents the HWF distribution histogram determined from gray matter voxels in whole brain across the entire group of subjects (n = 161), with an input noise level *ε*0 of 0.1, 0.2, 0.3 and 0.4 using CEEMD as well as EMD respectively. The histograms of HWF of IMF1 to IMF5 were colored by red, green, blue, magenta and cyan respectively. The heights of the histograms represent the number of voxels whose HWF equals to the frequency on the horizontal axis.

The histograms of the HWF distributions were presented in Fig 1, demonstrating the first five IMFs of the voxels in the whole-brain gray matter at different input noise level *ε*0 using the CEEMD method (Fig 1a–1d) or EMD (Fig 1e) approach across all the subjects. Each of the five histograms in Fig 1a–1e represents the statistic of the whole-brain gray matter voxels within distinct frequency bands, respectively. Considering the very similarity of the frequency content of different voxels at different sites of the brain (and subjects), the same IMF (IMFs, s = 1, 2, 3, 4 or 5) from all of the voxels approximately fell into the same frequency band. Consistent with a previous study [11], these five IMF components were derived to cover a frequency range from 0 to 0.22 Hz, with each interval range.

**Fig 2:**
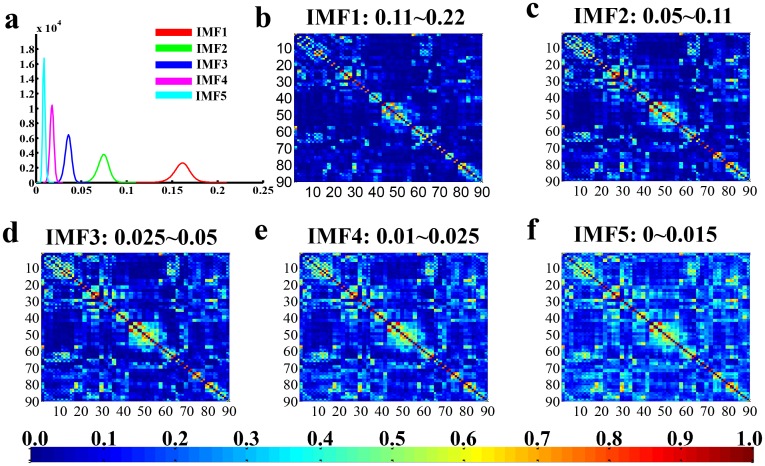
Schematic of frequency distribution and specificity of functional connectivity networks. Fig 2a represents the histograms of HWF of IMF1 to IMF5 using CEEMD (n = 161, *ε*_0_ = 0.04), which is the same as Fig 1d. These IMFs occupy different frequency bands in a descending order (IMF1: 0.11–0.22 Hz; IMF2: 0.05–0.11 Hz; IMF3: 0.025–0.05 Hz; IMF4: 0.01–0.025 Hz; IMF5: 0–0.015 Hz, respectively). Fig 2b–2f denote the group-mean inter-regional correlation matrices of each IMF component (AAL template, 90×90 correlation matrix, only the positive value was presented), and the number from 1 to 90 represents the corresponding ROI in AAL template, for details, refer to Table 1.

Referring to Fig 1a–1d, a high noise level was associated with relatively better concentrated intra-frequency bands and separated inter-frequency bins. Thus, the observed best performance in dividing the BOLD signals into five IMF components was demonstrated in Fig 1d. The presented results were acquired with an input noise level *ε*0 = 0.4. In addition, the consequences of other conditions (*ε*0 = 0.1, 0.2, 0.3) were provided as the supplementary materials (S1 Fig). As shown in Fig 1d, the frequency of each IMF fell into a unique frequency band, with the first IMF (IMF1) indicating the highest frequencies from 0.11to 0.22 Hz, IMF2 for 0.05 to 0.11 Hz, IMF3 for 0.025 to 0.05 Hz, IMF4 for 0.01 to 0.025 Hz, and IMF5 for the lowest frequency band from 0 to 0.015 Hz. Meanwhile, the histograms of HWF distributions derived from the EMD method was presented in Fig 1e to demonstrate the frequency bands or mode mixing effects. The FC matrices of each IMF component were displayed in Fig 2b–2f to represent the inter-regional FC subtended by time series components in the frequency bands defined by IMF 1–5. The results suggest that CEEMD can adaptively decompose the original time series into different intrinsic oscillatory modes that can be classified into distinctive frequency bands. Thus, CEEMD can be applied as a non-stationary and non-linear neurological signal processing method.

### Frequency dependent small world networks

**Fig 3:**
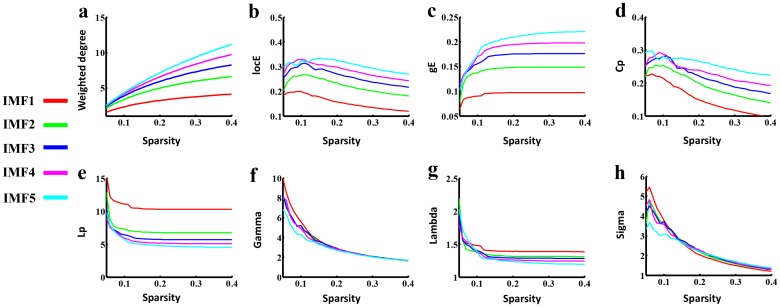
Small world properties in the frequency specific FCNs. From Fig 3a to Fig 3e, each figure shows the plot of global topological patterns of distinct frequency intervals (y-axis) versus sparsity (x-axis), including the weighted degree, local network efficiency (locE), global network efficiency (gE), mean clustering coefficient (Cp) and shortest path length (Lp) respectively. The ratio Gamma (Fig 3f) and Lambda (Fig 3g) of five frequency specific FCNs showed a much higher Cp and identical Lp value, compared with closely matched random networks across much sparsity. The saliency of small-worldness, Sigma, dynamically covered different frequency bands at various density intervals. Specifically, small world architecture is prominent in the IMF1, IMF3 and IMF5 component at a range of density threshold from 0.05 to 0.12, 0.12 to 0.18, and 0.24 to 0.4, respectively.

Previous studies have demonstrated that the small-world topology exists in large-scale brain functional and structural networks in humans [1, 32], in which the shortest path length between any pair of nodes is approximately equivalent to a comparable random network, but with greater local interconnectivity than a random network [28]. In the current study, the small world properties were investigated in each frequency specific brain networks. As shown in Fig 3, all five FCNs showed small-world architecture with more locally clustered (γ>1, Fig 3f) but almost identical path length (λ≈1, Fig 3g) over a wide range of densities. Inconsistent with Achard’s study [15], the small-worldness of these five FCNs was compared at multi-density and identified to be frequency-sparsity dependent. The saliency of small-worldness mainly covered three frequency bands at distinct density intervals. Specifically, small world architecture was prominent in the IMF1, IMF3and IMF5 components (Fig 3h). The mean clustering coefficient increased from IMF1 to IMF5 across most density intervals (Fig 3d), while the characteristic path length exhibited an opposite variation (Fig 3e). Additionally, network weighted degree or efficiency analysis demonstrated that lower frequency bands were associated with higher weighted degree or global or local network efficiency (Fig 3a–3c). The results also suggested that high-frequency band IMF1 exhibited small world properties, which may be discarded in conventional FC analysis.

### Spatial distribution of hub regions in distinct frequency bands

**Fig 4:**
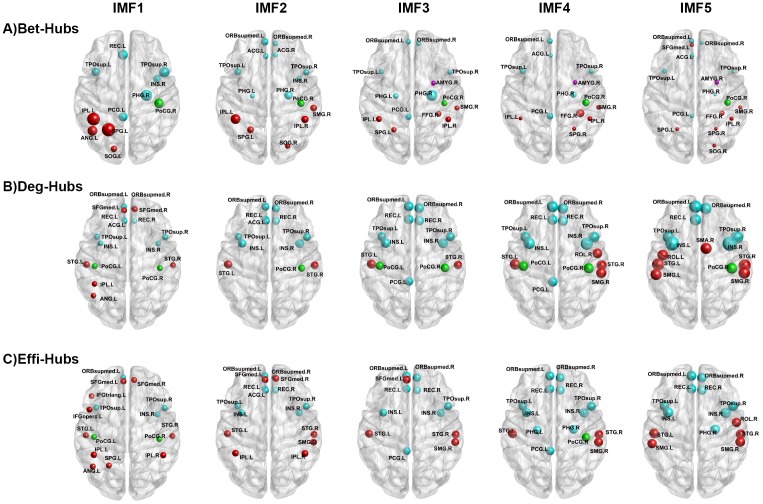
The spatial distribution of hub regions. Three dimensional rendering maps show hub regions defined by nodal betweenness (A), nodal weighted degree (B), and nodal efficiency (C) in five IMFs. The hub nodes shown in red, green, cyan and magenta color donate Associations, Primary, Paralimibic and Subcortical regions respectively as described by Achard et al. (2006) The size of the node represents their nodal topological characteristics. Hub regions are visualized using the BrainNet viewer (NKLCNL, Beijing Normal University). For the abbreviations of the regions, refer to Table 1.

The difference was observed extensively in the frequency dependent global topological patterns in brain networks, therefore, it is hypothesized that the nodal characteristics may vary in the frequency specific brain networks. Nevertheless the spatial distribution of hubs defined by nodal betweenness, weighted degree or nodal efficiency was similar across different frequency bands (Fig 4). Consistent with previous studies [15, 25, 31, 36–38], all the hubs are mainly concentrated on association, primary and paralimbic cortex. Remarkably, low frequency interval, particularly smaller than 0.1 Hz, is under investigation in previous FC studies [4, 5, 39]. On the contrary, in the present study, consistent spatial distribution of hub regions was observed in both low frequency (< 0.1 Hz) bands and higher frequency bands (IMF1 > 0.1 Hz). Fig 4 provided the 3D representations of the hub distributions to visualize these hubs in distinct frequency intervals. In addition, the detailed value of nodal topological characteristics in each frequency specific brain networks were listed in S1, S2, S3 Tables.

### Reproducibility of the findings

**Fig 5:**
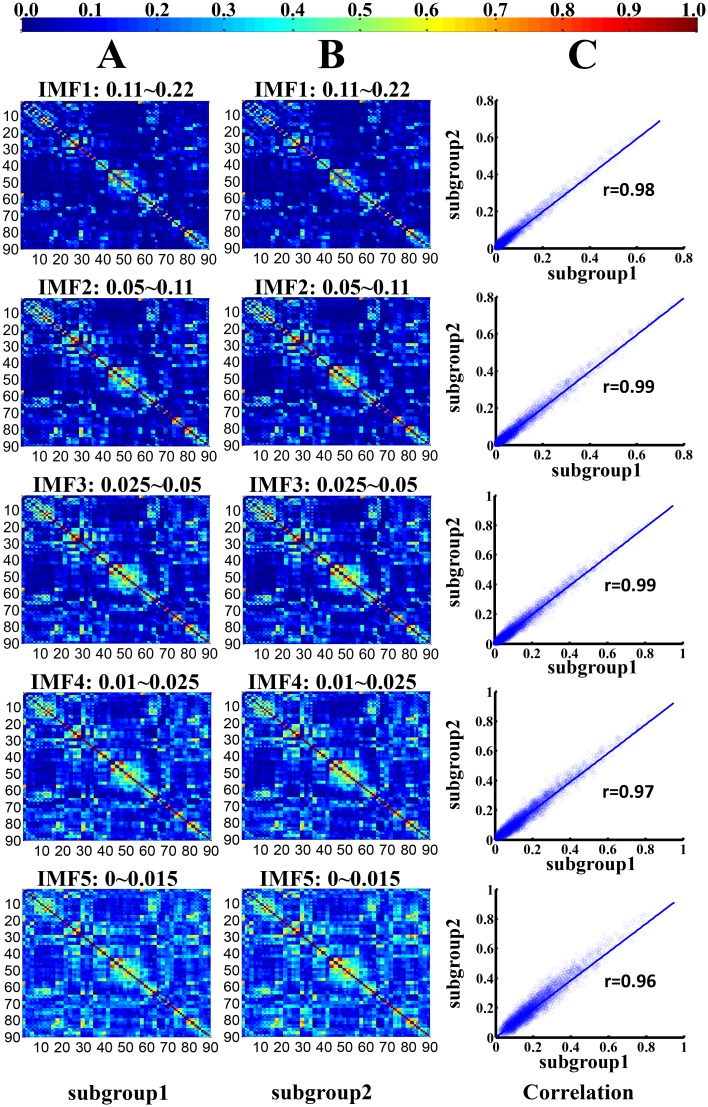
Split-half reproducibility of the frequency dependent functional connectivity weighted networks. (A) and (B) represent the group-mean frequency specific FC weighted networks of two specific subgroups in each IMF component. (C) denotes the corresponding correlation maps between each pair of frequency specific FCNs in the two subgroups.

**Fig 6:**
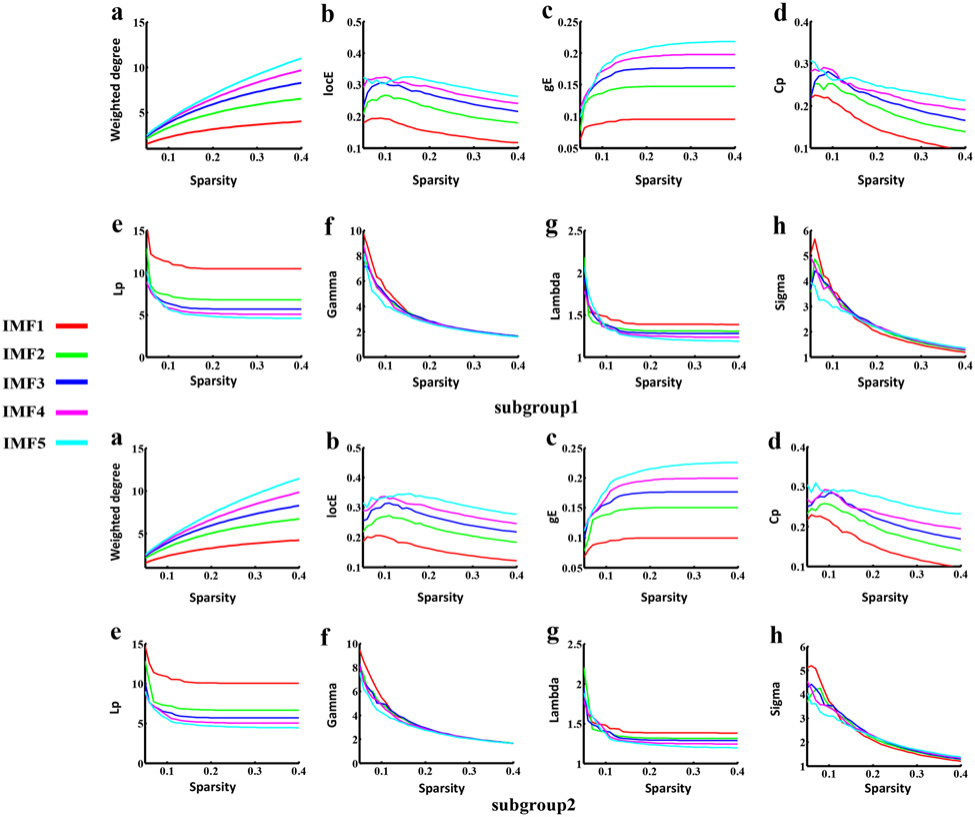
Split-half reproducibility of global topological patterns in both subgroups. From top to bottom, subgroup1 and subgroup2 were presented respectively. Both subgroups had the similar patterns with the whole group results (Fig 3), showing that ultra-low frequency bands (IMF5) have both salient local and global connectivity patterns. The saliency of small-worldness dynamically covered different frequency bands at various density intervals.

**Fig 7:**
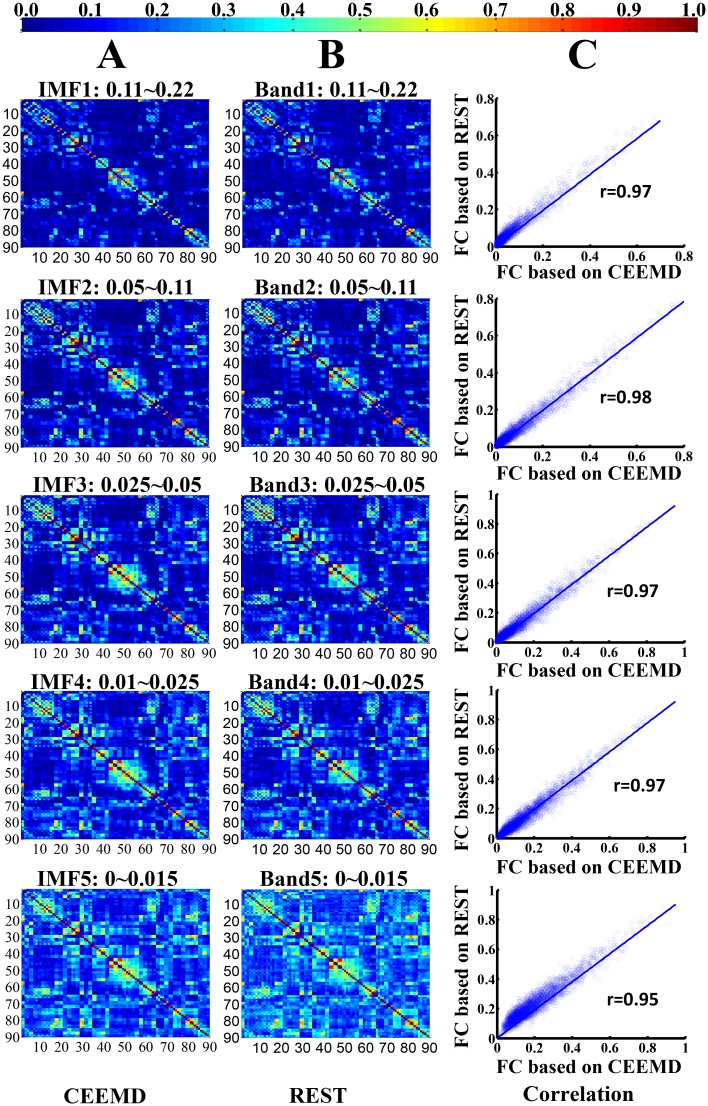
Comparison between the CEEMD and REST methods. (A) and (B) represent the group-mean frequency specific FC weighted networks of these two methods, namely CEEMD in current study and conventional rectangular window band-pass filter (REST), in each IMF component. (C) denotes the corresponding correlation maps between each pair of frequency specific FCNs using the two methods.

**Fig 8:**
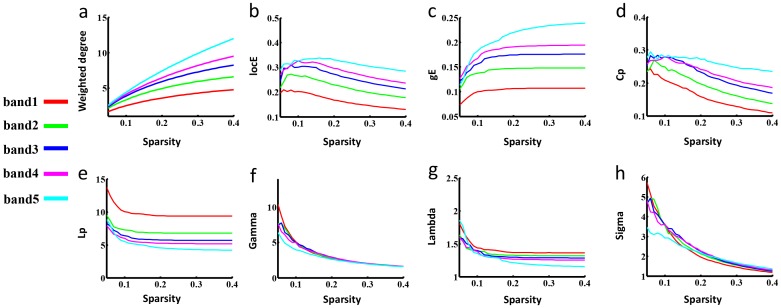
Small world properties in the frequency specific FCNs using REST. Fig 8 show the plot of global topological patterns of distinct frequency intervals (y-axis) separated by a conventional ideal rectangular window band-pass filter versus sparsity (x-axis). The meaning of these figures is the same as in Fig 3.

To test the stability of brain FCNs construction and the corresponding topological properties, split-half reliability was performed by dividing all participants into two independent subgroups. Visual examination indicated that the FC patterns of each IMF were similar between the two datasets (Fig 5A & 5B) and in the aforementioned whole group (Fig 2). Further statistical analyses (IMF1: r = 0.98, *P* < 0.0001; IMF2: r = 0.99, *P* < 0.0001; IMF3, r = 0.99, *P* < 0.0001; IMF4, r = 0.97, *P* < 0.0001; IMF5, r = 0.96, *P* < 0.0001) revealed a significant inter-group correlation between the mean FCNs of each IMF (Fig 5C). In addition, the global topological patterns of each frequency band were calculated in both subgroups (Fig 6) to demonstrate similar patterns with whole group analysis (Fig 3). The comparison between CEEMD and REST also indicated the effectiveness of CEEMD and the rational specific frequency bands in each IMF component. Visual inspections indicated that the FC patterns of each specific frequency band were similar within either CEEMD or REST (Fig 7A & 7B). Further statistical analyses (0.11–0.22 Hz, r = 0.97, *P* < 0.0001; 0.05 to 0.11 Hz, r = 0.98, *P* < 0.0001; 0.025 to 0.05Hz, r = 0.97, *P* < 0.0001; 0.01 to 0.025 Hz, r = 0.97, *P* < 0.0001; 0–0.015 Hz, r = 0.95, *P* < 0.0001) revealed a significant correlation in the mean FCNs of each frequency bin between the two methods (Fig 7C). In addition, the global topological patterns of frequency specific FCNs were calculated by using a rectangular window to perform band-pass filtering (Fig 8).

## Discussion

Previous studies [40, 41] have suggested that numerous brain oscillations are well organized into several brain rhythms in support of complex brain activities within distinct frequency bands. These rhythms could temporally coexist in the same or different brain areas and may interact with each other with specific properties and physiological functions [40, 41]. In the current study, a data driven method CEEMD was applied to separate these inherent brain oscillations embedded in BOLD signals. Combined with graph theory, our investigation revealed three main findings: (i) CEEMD is a more efficient method in separating the intrinsic coexisting rhythms within distinct frequency bands than EMD [11] due to its capability in eliminating mode-mixing effects. Results indicate that the five frequency bins defined by our methods may be justifiable division of the full frequency band of BOLD signals that are distinct from the sub-frequency intervals derived from the scale-free dynamics of brain activities by Buzsaki et al [40]; (ii) at the global topological level, results revealed that several global topological properties, including network weighted degree, network efficiency, Cp and Lp, are prominent in the ultra-low frequency bands from 0 to 0.015 Hz, while the saliency of small-worldness is frequency-sparsity dependent; and (iii) at the nodal topological level, the spatial distribution of hubs defined by nodal betweenness, weighted degree or nodal efficiency was similar across the five brain oscillations.

By using graph theory analysis, the results of global topological patterns in distinct frequency bands (Fig 3) indicate that a higher FC is associated with lower frequency bands. The results are consistent with previous studies [9, 10, 42], suggesting that the frequency characteristics of FC are tightly associated with the corresponding inter-regional physical distance. Specifically, the correlations in long-distance brain regions are concentrated within ultra-low frequencies (0.01–0.06 Hz), but over a wider frequency range (0.01–0.14 Hz) within short-distance brain regions [10]. Thus, these results further highlight the efficacy of the CEEMD method in isolating the frequency bands of BOLD signals.

With regard to the sigma measure, results suggest that the saliency of small world networks is frequency-sparsity dependent (Fig 3h). Particularly, it is emphasized that to compare the sigma across different frequency bands by calculating the AUC for the sigma measure is not acceptable due to the determination of the interest sparsity interval. Considering the selection of the sparsity intervals from 0.05 to 0.15 or from 0.05 to 0.25 or from 0.05 to 0.4 to estimate the value of AUC, the small-worldness of IMF1, IMF3 and IMF5 will be salient respectively. Moreover, these results were tested-retested in the current study (Figs 6 and 8, S1, S2, S3 Figs). In addition, a previous study (Achard *et al*.[15]) indicate that the small-world network is most salient in the 0.03–0.06 Hz interval, corresponding to the frequency band of IMF3 in the current study. Inconsistent with Achard *et al*.[15], the small-worldness was calculated at multi-density in the current study, while it was estimated at only one threshold value previously.[15]. Since there currently is no formal consensus in regard to the selection of thresholds, the phenomenon of the frequency-sparsity dependent small-worldness was observed in our data. It is conjectured that all five frequency bands may possess significance to understand our brain function in the context of specific conditions (e.g., sparsity). Salient small world properties demonstrated by fMRI in the high frequency band (IMF1, 0.11–0.22) seem to be caused by susceptibility artifacts, since the frequency band such as IMF1 is covered by respiratory frequency interval from 0.1 to 0.5 Hz [43]. However, previous studies have suggested that the spectral range of BOLD signals greater than 0.1 Hz demonstrate consistent patterns with low-frequency fluctuations (< 0.1 Hz) [19, 44]. In addition, high-frequency FC is concentrated in local brain regions, which may lead to a prominent value of γ = Cp_(real)_/Cp_(rand)_ in IMF1 than other IMF in the context of relatively sparse FCN [9, 10, 42]. Conclusively, the findings in the current study, on one hand, indicate that different sub-frequency bands require more attention other than single frequency intervals; on the other hand, challenge the notion that FCNs of resting state fMRI are simple “low frequency” spontaneous signal fluctuations.

Considering the spatial patterns of hub regions, the results of similar distribution of hubs in five frequency intervals indicate that these five sub-bands may possess approximately the same long-range spatial connectivity patterns. Combined with the results of binary FCNs (S2 Fig), it may be speculated that distinct frequency bands mainly influence the weighted coefficients but slightly effect the spatial long-range connectivity attributes. Future studies are required to validate the hypothesis and to discuss the difference of short-range connectivity patterns in distinct frequency bins (e.g., modularity and network motifs) [45]. Remarkably, similar hubs in higher frequency intervals IMF1 further suggest that the FCN of resting state fMRI in this specific frequency band may carry some useful physiological information.

### Methodology consideration

In the FC analysis in BOLD fMRI, there is no consensus on the necessity to correct global signals in fMRI time courses [46]. One previous study [46] suggested that without global signal correction, nodes along the inter-hemispheric fissure would be highly connected while some nodes and subgraphs around white-matter tracts would become disconnected from the rest of the network. In the present study, on one hand, the discussion of regression with or without global signal is out of our scope; on the other hand, the consequences of topological patterns without regressing out the global signal were provided in supplementary materials (S3 and S4 Figs, S4, S5, S6 Tables). Results showed that the variation trends of topological patterns among five specific frequencies were not influenced by the regression of global signals. In addition, typical graph analyses of weighted networks ignored negative ties while recent studies proposed to incorporate negative weights into analyses of subgraph detection. Here, we followed the traditional approach.

### Limitations

The present study should be considered as a preliminary study to investigate the frequency specificities of brain networks and has a few limitations. The influence of head motion on the frequency specificities in the small world network was not discussed, because a number of recent studies [47, 48] have reported decreased long range connectivity and increased local connectivity due to head motion. Thus, head motion is required to be concerned in our further study. In addition, the analysis of the node definition was limited to AAL template-based brain networks. A previous study suggest that the topological organization of brain networks may be affected according to the different parcellation strategies applied [49]. Future studies will be needed to clarify the difference resulting from various node definitions. Moreover, short-range connectivity patterns such as modularity or motif were not considered in the current study, and future efforts integrating the findings from other network parameters will provide valuable additions to our observations. Last but not least, the highest frequency in this study is smaller than 0.25 Hz, however, higher frequency fMRI data can be generated by using the most recently developed technology multiband echo planar imaging [50]. Future efforts are required to investigate the potential applications of combining CEEMD with multiband echo planar imaging.

## Conclusion

In the current study, we have introduced a novel method CEEMD to divide the resting state fMRI signals into five specific oscillations within distinct frequency bands, and have shown how these can be used to explore the frequency characteristics in resting state brain networks for the first time. Our results showed evidence that several global topological properties, including the network weighted degree, network efficiency, Cp and Lp, are prominent in the ultra-low frequency bands from 0 to 0.015 Hz, while the saliency of small-worldness is frequency-sparsity dependent. The divergent frequency-specific topological connectivity characteristics are associated with distinct frequency-dependent FC, which may reflect the assorted cytoarchitecture of different brain areas or anatomical distance. Moreover, CEEMD may offer a novel approach to investigate the frequency specificities existing extensively within the BOLD signals. Combined with graph theory analysis, the frequency specific topological organizations of brain networks are well investigated. Most importantly, future direction toward the frequency specific brain networks may focus on elucidating the relationship between the frequency specific topological profiles and cognitive performance or psychiatric brain disorders, opening up new avenues to better understanding the human brain.

## Supporting Information

S1 Fig Results of global topological patterns using the CEEMD method at different input noise levels. From top to bottom, figures showed the global topological patterns with the input noise level *ε*0 equals to 0.1, 0.2 and 0.3, respectively. The global topological patterns shown here were similar with these in the condition of *ε*0 = 0.4.
(TIF)

S2 Fig Small world properties of frequency specific functional connectivity binary networks. **a,** Network degree increases as the sparsity is increased, and five IMFs are equal at each sparsity. **b and c,** The mean clustering coefficient (Cp) and shortest path length of these binary FCNs appear to lost the regular variation tendency compared with frequency specific weighted FCNs. **d, e and f,** here, the ratio *γ* and small-worldness *σ* in binary FCNs tend to have similar results with weighted FCNs, which demonstrated the small-worldness to be salient in frequency bins of IMF1, IMF3 and IMF5.
(TIF)

S3 Fig Small world properties of frequency specific brain networks without regression of global signal. The global topological patterns were similar with these regressed out the global signals, demonstrating that small-worldness *σ* is salient in IMF1, IMF3, and IMF5 components at different densities. This results are inconsistent with that described by Achard et al. (2006) and Xia Liang et al. (2012).
(TIF)

S4 Fig The spatial distribution of hub regions without regression of the global signal. Three dimensional rendering maps show hub regions defined by nodal betweenness (A), nodal weighted degree (B), and nodal efficiency (C) in five IMFs. The hub nodes shown in red, green, cyan and magenta color refer to Associations, Primary, Paralimibic and Subcortical regions respectively as described by Achard et al. (2006), and the size of the nodes represents their nodal topological characteristics. Hub regions were visualized using the BrainNet viewer (NKLCNL, Beijing Normal University). For the abbreviations of the regions, refer to Table 1.
(TIF)

S1 File Empirical mode decomposition. (PDF)

S1 TableBetweenness-based hub regions with global signal regression.The frequency-specific brain networks for each participants were constructed using an AAL template. The hub regions based on regional betweenness were identified if ${\text{B}}_{\text{i}}^{\text{(w,auc)}}$ was at least 1 SD greater than the mean ${\text{B}}_{\text{i}}^{\text{(w,auc)}}$ of the network. The hubs were then sorted by the corresponding AUC values in each IMF. The cortical regions were classified as primary, association, and paralimbic.(DOCX)

S2 TableDegree-based hub regions with global signal regression.The frequency-specific brain networks for each participants were constructed using an AAL template. The hub regions based on regional weighted degree were identified if ${\text{S}}_{\text{i}}^{\text{(w,auc)}}$ was at least 1 SD greater than the mean ${\text{S}}_{\text{i}}^{\text{(w,auc)}}$ of the network. The hubs were then sorted by the corresponding AUC values in each IMF. The cortical regions were classified as primary, association, and paralimbic.(DOCX)

S3 TableEfficiency-based hub regions with global signal regression.The frequency-specific brain networks for each participants were constructed using an AAL template. The hub regions based on regional efficiency were identified if ${\text{E}}_{\text{i,glob}}^{\text{(w,auc)}}$ was at least 1 SD greater than the mean ${\text{E}}_{\text{i,glob}}^{\text{(w,auc)}}$ of the network. The hubs were then sorted by the corresponding AUC values in each IMF. The cortical regions were classified as primary, association, and paralimbic.(DOCX)

S4 TableBetweenness-based hub regions without global signal regression.The frequency-specific brain networks for each participants were constructed using an AAL template. The hub regions based on regional betweenness were identified if ${\text{B}}_{\text{i}}^{\text{(w,auc)}}$ was at least 1 SD greater than the mean ${\text{B}}_{\text{i}}^{\text{(w,auc)}}$ of the network. The hubs were then sorted by the corresponding AUC values in each IMF. The cortical regions were classified as primary, association, and paralimbic.(DOCX)

S5 TableDegree-based hub regions without global signal regression.The frequency-specific brain networks for each participants were constructed using an AAL template. The hub regions based on regional weighted degree were identified if ${\text{S}}_{\text{i}}^{\text{(w,auc)}}$ was at least 1 SD greater than the mean ${\text{S}}_{\text{i}}^{\text{(w,auc)}}$ of the network. The hubs were then sorted by the corresponding AUC values in each IMF. The cortical regions were classified as primary, association, and paralimbic.(DOCX)

S6 TableEfficiency-based hub regions without global signal regression.The frequency-specific brain networks for each participants were constructed using an AAL template. The hub regions based on regional efficiency were identified if ${\text{E}}_{\text{i,glob}}^{\text{(w,auc)}}$ was at least 1 SD greater than the mean ${\text{E}}_{\text{i,glob}}^{\text{(w,auc)}}$ of the network. The hubs were then sorted by the corresponding AUC values in each IMF. The cortical regions were classified as primary, association, and paralimbic.(DOCX)
