# Supplementary figures and images for: Frequency Dependent Topological Patterns of Resting-State Brain Networks
Source: PLoS One. 2015 Apr 30;10(4):e0124681. doi: 10.1371/journal.pone.0124681 (PMC4415801; doi:10.1371/journal.pone.0124681)

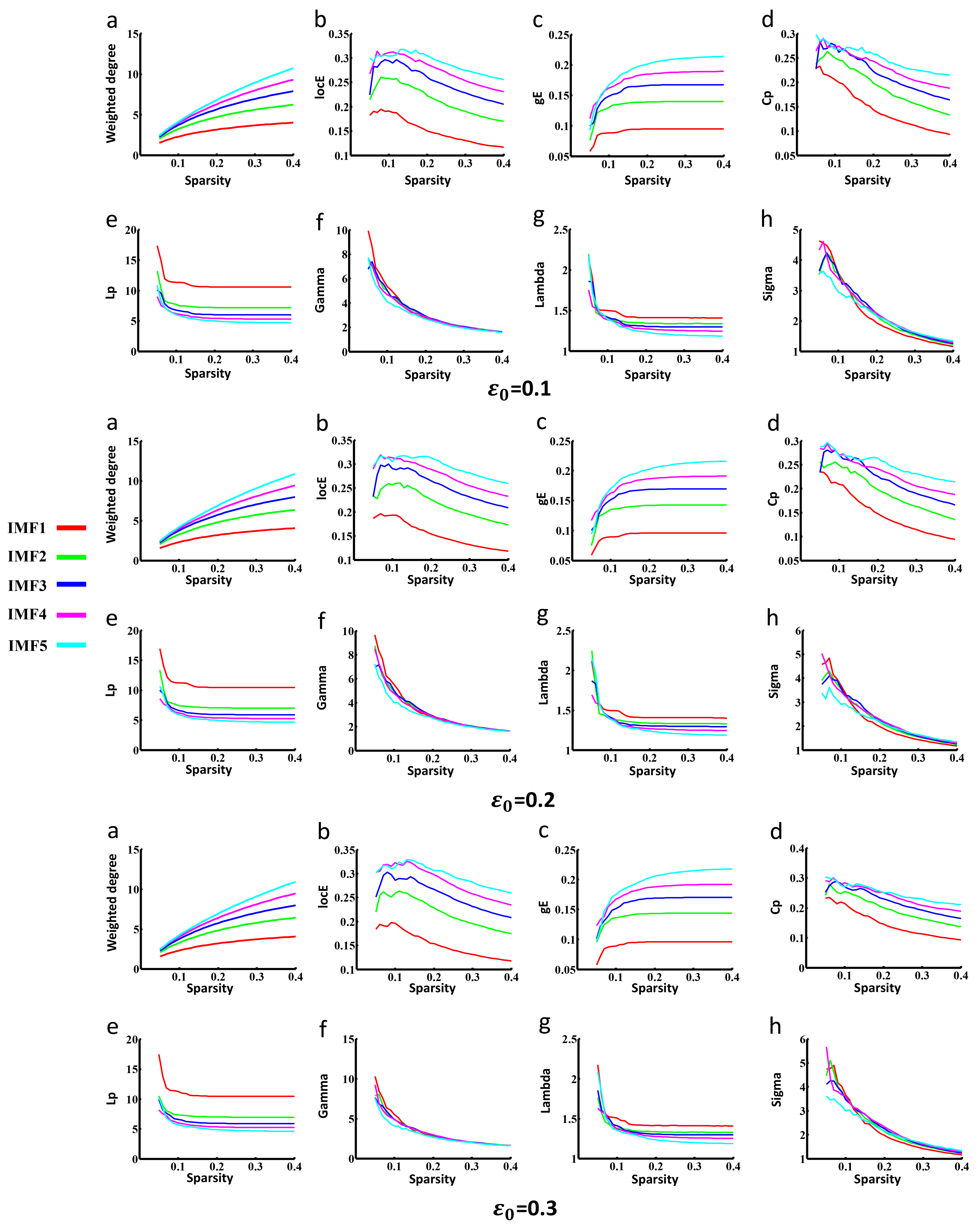

Supplement: S1 Fig — From top to bottom, figures showed the global topological patterns with the input noise level ε0 equals to 0.1, 0.2 and 0.3, respectively. The global topological patterns shown here were similar with these in the condition of ε0 = 0.4. (TIF) [file pone.0124681.s001.tif]

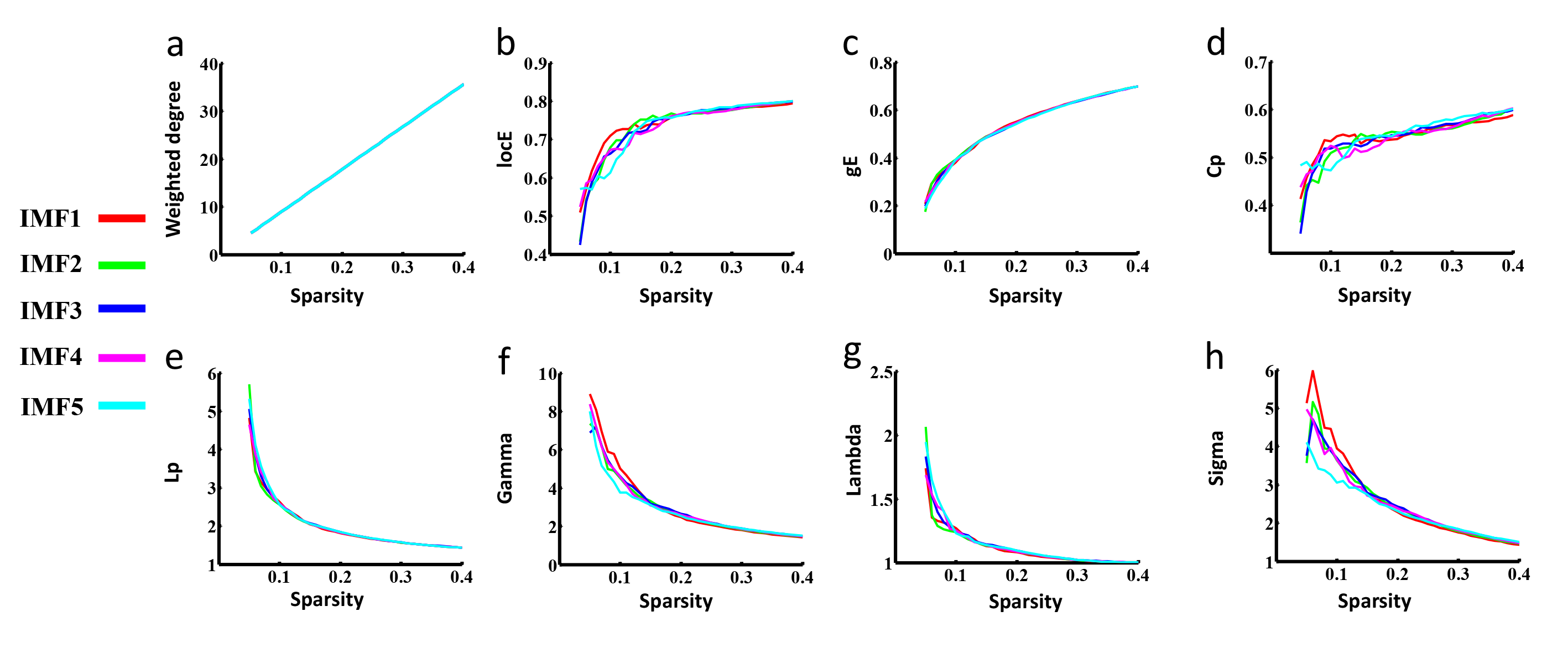

Supplement: S2 Fig — a, Network degree increases as the sparsity is increased, and five IMFs are equal at each sparsity. b and c, The mean clustering coefficient (Cp) and shortest path length of these binary FCNs appear to lost the regular variation tendency compared with frequency specific weighted FCNs. d, e and f, here, the ratio γ and small-worldness σ in binary FCNs tend to have similar results with weighted FCNs, which demonstrated the small-worldness to be salient in frequency bins of IMF1, IMF3 and IMF5. (TIF) [file pone.0124681.s002.tif]

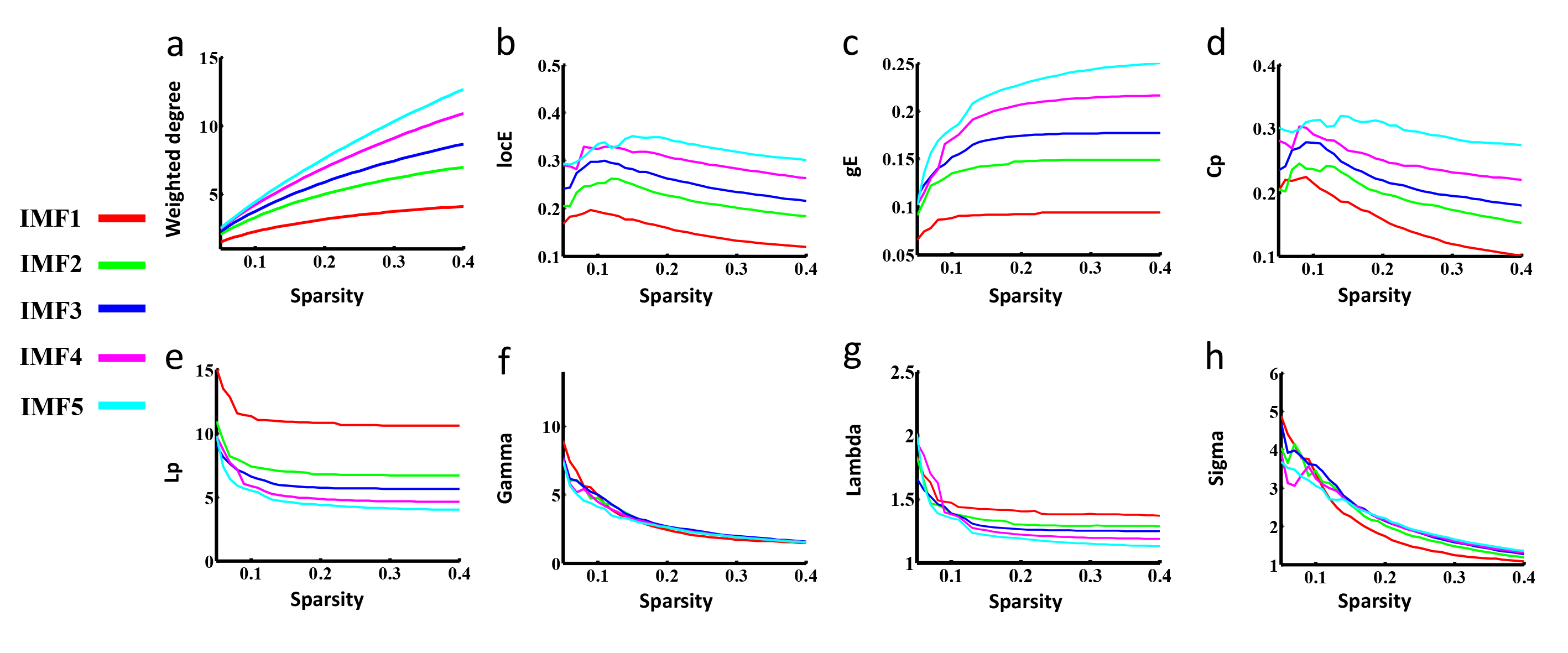

Supplement: S3 Fig — The global topological patterns were similar with these regressed out the global signals, demonstrating that small-worldness σ is salient in IMF1, IMF3, and IMF5 components at different densities. This results are inconsistent with that described by Achard et al. (2006) and Xia Liang et al. (2012). (TIF) [file pone.0124681.s003.tif]

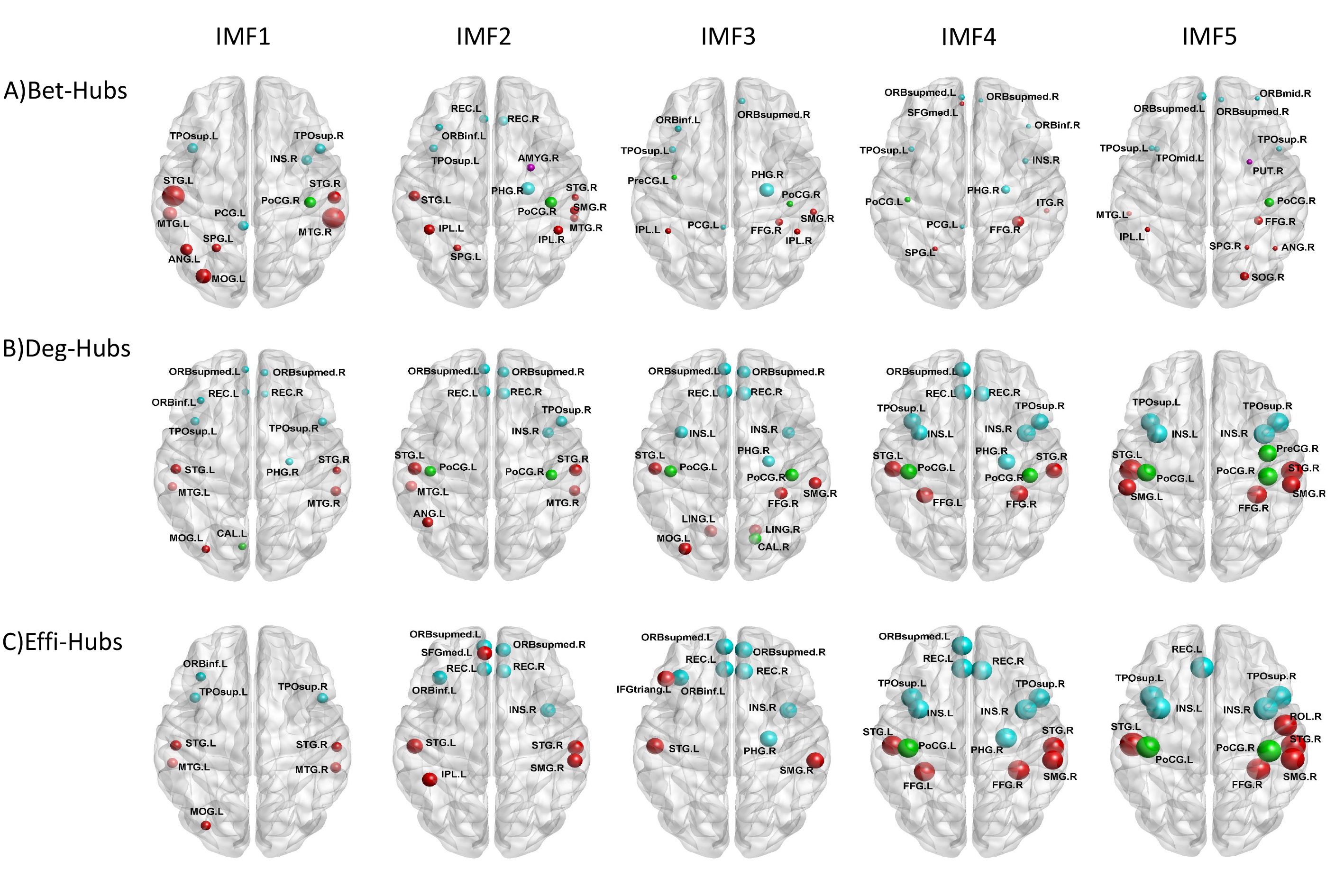

Supplement: S4 Fig — Three dimensional rendering maps show hub regions defined by nodal betweenness (A), nodal weighted degree (B), and nodal efficiency (C) in five IMFs. The hub nodes shown in red, green, cyan and magenta color refer to Associations, Primary, Paralimibic and Subcortical regions respectively as described by Achard et al. (2006), and the size of the nodes represents their nodal topological characteristics. Hub regions were visualized using the BrainNet viewer (NKLCNL, Beijing Normal University). For the abbreviations of the regions, refer to Table 1. (TIF) [file pone.0124681.s004.tif]
