# Supplementary material for: Frequency Dependent Topological Patterns of Resting-State Brain Networks
Source: PLoS One. 2015 Apr 30;10(4):e0124681. doi: 10.1371/journal.pone.0124681 (PMC4415801; doi:10.1371/journal.pone.0124681)
Supplement: S1 File — (PDF) [file pone.0124681.s005.pdf]

Supplementary Information

Empirical mode decomposition

The whole procedure of EMD followed Huang [1]. Distinct from most of the data analysis methods, EMD is an adaptive and efficient method to decompose nonlinear and non-stationary biomedical signals [2],which extracts a series of IMFs from the analyzed signal shifting stage by stage. Mathematically, for a real-valued BOLD signal $x(t)$, the standard EMD determines a set of N IMFs { $IMF_i(t)$ }, i = 1 to N, and a monotonic residue signal $r(t)$, so that

$$x(t) = \sum_{i=1}^{N} IMF_i(t) + r(t) \qquad \text{(Eq.1)}$$

To ensure the yield of meaningful frequency estimates by the time frequency spectra (e.g. no negative frequencies), all the IMFs satisfied the following conditions: the number of zero crossings and the number of extrema equaled or differed at most by one; and the mean value of the upper and lower envelopes defined by the local maxima and local minima was zero at all points. With the above definition of IMF, all the signals could be decomposed in the following steps:

1) Identify all the local extrema;

2) Interpolate all the minima (resp. maxima) to produce the lower (resp. upper) signal envelope, $elow(t)$ (resp. $\mathrm{eup}(t)$);

3) To obtain the local mean time course using $\mathrm{m(t)}=\left[\mathrm{elow}(t)+\mathrm{eup}(t)\right]/2$;

4) Obtain the "oscillatory mode" from $r(t) = x(t) - m(t)$;

5) If $r(t)$ meets the standard stopping criterion (sifting process only after the IMF condition is achieved for S consecutive times, in the current study S=3), $IMF_i(t) = r(\mathrm{t})$ becomes an IMF. Otherwise set $x(t) = r(t)$ and repeat the above steps.

6) To obtain the next IMF by regarding the residue $r(\mathrm{t})$ as a new data and repeating the same procedure

until $r(\mathrm{t})$ was smaller than a predetermined value, or $r(\mathrm{t})$ became a monotone function, the shifting process was terminated, or else repeated as the last step. Thus, a series of IMFs were obtained.

Reference

1. Huang, N.E., et al., *The empirical mode decomposition and the Hilbert spectrum for nonlinear and non-stationary time series analysis.* Proceedings of the Royal Society of London. Series A: Mathematical, Physical and Engineering Sciences, 1998. **454**(1971): p. 903-995.
2. Lin, C.-F. and J.-D. Zhu, *Hilbert–Huang transformation-based time-frequency analysis methods in biomedical signal applications.* Proceedings of the Institution of Mechanical Engineers, Part H: Journal of Engineering in Medicine, 2012. **226**(3): p. 208-216.
