# Supplementary material for: Frequency Dependent Topological Patterns of Resting-State Brain Networks
Source: PLoS One. 2015 Apr 30;10(4):e0124681. doi: 10.1371/journal.pone.0124681 (PMC4415801; doi:10.1371/journal.pone.0124681)
Supplement: S6 Table — The frequency-specific brain networks for each participants were constructed using an AAL template. The hub regions based on regional efficiency were identified if Ei,glob(w,auc) was at least 1 SD greater than the mean Ei,glob(w,auc) of the network. The hubs were then sorted by the corresponding AUC values in each IMF. The cortical regions were classified as primary, association, and paralimbic. (DOCX) [file pone.0124681.s011.docx]

## S6 Table. Efficiency-based hub regions without global signal regression

S6.1 Table. Hub regions in IMF1 component.

| Regions | Class | Nodal global efficiency (AUC) |
| --- | --- | --- |
| MTG.R | Association | 0.0328 |
| STG.L | Association | 0.0326 |
| TPOsup.L | Paralimbic | 0.0320 |
| TPOsup.R | Paralimbic | 0.0319 |
| MTG.L | Association | 0.0314 |
| STG.R | Association | 0.0310 |
| MOG.L | Association | 0.0298 |
| ORBinf.L | Paralimbic | 0.0292 |

S6.2 Table. Hub regions in IMF2 component.

| Regions | Class | Nodal global efficiency (AUC) |
| --- | --- | --- |
| STG.L | Association | 0.0476 |
| STG.R | Association | 0.0465 |
| ORBsupmed.L | Paralimbic | 0.0451 |
| INS.R | Paralimbic | 0.0445 |
| SMG.R | Association | 0.0445 |
| REC.L | Paralimbic | 0.0445 |
| REC.R | Paralimbic | 0.0445 |
| IPL.L | Association | 0.0444 |
| ORBsupmed.R | Paralimbic | 0.0443 |
| SFGmed.L | Association | 0.0441 |
| ORBinf.L | Paralimbic | 0.0440 |

S6.3 Table. Hub regions in IMF3 component.

| Regions | Class | Nodal global efficiency (AUC) |
| --- | --- | --- |
| REC.R | Paralimbic | 0.0525 |
| REC.L | Paralimbic | 0.0522 |
| ORBsupmed.R | Paralimbic | 0.0520 |
| ORBsupmed.L | Paralimbic | 0.0518 |
| STG.L | Association | 0.0512 |
| SMG.R | Association | 0.0510 |
| PHG.R | Paralimbic | 0.0510 |
| ORBinf.L | Paralimbic | 0.0509 |
| IFGtriang.L | Association | 0.0508 |
| INS.R | Paralimbic | 0.0507 |

S6.4 Table. Hub regions in IMF4 component.

| Regions | Class | Nodal global efficiency (AUC) |
| --- | --- | --- |
| INS.R | Paralimbic | 0.0636 |
| FFG.R | Association | 0.0628 |
| PHG.R | Paralimbic | 0.0627 |
| STG.L | Association | 0.0626 |
| REC.L | Paralimbic | 0.0621 |
| TPOsup.R | Paralimbic | 0.0617 |
| REC.R | Paralimbic | 0.0616 |
| STG.R | Association | 0.0616 |
| TPOsup.L | Paralimbic | 0.0608 |
| FFG.L | Association | 0.0606 |
| PoCG.L | Primary | 0.0605 |
| INS.L | Paralimbic | 0.0604 |
| SMG.R | Association | 0.0604 |
| ORBsupmed.L | Paralimbic | 0.0602 |

S6.5 Table. Hub regions in IMF5 component.

| Regions | Class | Nodal global efficiency (AUC) |
| --- | --- | --- |
| STG.L | Association | 0.0745 |
| STG.R | Association | 0.0727 |
| TPOsup.R | Paralimbic | 0.0718 |
| INS.R | Paralimbic | 0.0711 |
| INS.L | Paralimbic | 0.0698 |
| PoCG.R | Primary | 0.0695 |
| SMG.R | Association | 0.0694 |
| TPOsup.L | Paralimbic | 0.0689 |
| FFG.R | Association | 0.0686 |
| PoCG.L | Primary | 0.0680 |
| REC.L | Paralimbic | 0.0674 |
| ROL.R | Association | 0.0674 |

The frequency-specific brain networks for each participants were constructed using an AAL template. The hub regions based on regional efficiency were identified if $\text{E}_{\text{i,glob}}^{\text{(w,auc)}}$ was at least 1 SD greater than the mean $\text{E}_{\text{i,glob}}^{\text{(w,auc)}}$ of the network. The hubs were then sorted by the corresponding AUC values in each IMF. The cortical regions were classified as primary, association, and paralimbic.
